# Knowledge, Attitude and Practice of Emergency Contraceptives Among Adama University Female Students

**DOI:** 10.4314/ejhs.v20i3.69449

**Published:** 2010-11

**Authors:** Dejene Tilahun, Tsion Assefa, Tefera Belachew

**Affiliations:** 1 Department of Health Education and Behavioral Sciences Jimma University; 2 Department of population and Family Health, Jimma University

**Keywords:** emergency contraceptives, knowledge, attitude, practice, Ethiopia

## Abstract

**Background:**

Unwanted pregnancy followed by unsafe abortion is one of the major worldwide health problems, which has many negative consequences on the health and well-being of women. Information about women's knowledge, attitude and practice of emergency contraceptives plays a major role in the reduction of unwanted pregnancy; however, there are no studies about this issue in the study area. This study assessed Adama University female students' knowledge, attitude and practice of emergency contraceptives.

**Method:**

A cross-sectional study design was employed from February 1 to 30/2009, on 660 regular undergraduate female students of Adama University. Data were entered and analyzed using SPSS for windows version 16.0. Logistic regression was used to identify the association between variables and emergency contraceptive knowledge, attitude and practice. P-value less than 0.05 at 95% CI was taken for statistical significance.

**Results:**

Of the total, 660 respondents, 194(29.4%) were sexually active, 63(9.4%) had history of pregnancy and 49(7.4%) had history of abortion. About 309 (46.8%) of the students had heard about emergency contraceptives and from those who heard emergency contraceptives, 27.2% had good knowledge. Majority, four hundred fifteen (62.9%) of the students had positive attitude towards it. However, only 31(4.7%) had used emergency contraceptive methods.

**Conclusion:**

This study demonstrated lack of awareness, knowledge and utilization of emergency contraceptives among Adama University female students. Hence behavioral change strategies should be considered by responsible bodies to improve knowledge and bring attitudinal change on use of emergency contraception.

## Introduction

Higher Education students' unwanted pregnancies pose a major public health problems in the developed and developing countries(1,2,3) including Ethiopia(4,5) and are associated with far reaching effects such as jeopardizing students' educational progress and future careers (1–5). These pregnancies are mostly unplanned and unintended, and many are terminated either legally or illegally. Around 30%–50% of women presenting for choice on termination of pregnancy were not using contraceptives at the time of contraception, and similar numbers of pregnancies were unplanned and unwanted (1,2).

Unwanted pregnancy is a big problem in Ethiopia; more than 60% of the pregnancies in adolescents are unwanted which is an alarming figure, and most of these pregnancies particularly in adolescents end up with unsafe abortion (4). According to the survey conducted in 2000 by ESOG in nine administrative regions, 25.6% abortion cases were induced abortions. Among abortion cases, 60% were unplanned, and 50% were unwanted. Abortion related mortality was 1,209 per 100,000 live births (6, 7).

In about half of all unwanted pregnancies, conception occurs due to inadequate guidance to use contraception effectively, including the users' inability to address their feelings, poor attitudes towards contraceptives, and lack of motivations (2). Despite the Ethiopian government's effort to prevent unwanted pregnancies and abortion among youths of age less than 24 years, the number of youths requesting termination of pregnancy is increasing annually (4,8). Despite the availability of contraceptives with affordable costs, there is a large number youths' with unwanted pregnancies and unsafe abortion (4).

Different studies showed that the knowledge, attitude and practice of emergency contraception among women are limited (4, 6–9).

As there are no studies in this problem in the area, this study was conducted to assess knowledge, attitude and use of emergency contraceptives among female students of Adama University.

## Subjects and Methods

This study was conducted from February 1 to 30/2009 in Adama University, located 100 Kms Southeast of Addis Ababa. According to the information obtained from the Registrar Office of Adama University, there were 45 departments at different schools during the conduct of the study. The total number of students in the University was 11788, females accounted for 27.2%.

Institution based cross-sectional study design was employed among female students at Adama University. The sample size was calculated using single population proportion formula.

From those who had sexual experience, the proportion of students who practiced emergency contraceptive methods was assumed to be 73.4% (10). By adding non-response rate of 10%, and multiplying by a design effect of 2) a sample of 660 was determined.

A two-stage sampling technique was used; where first 25 departments were selected from the total 45 departments using lottery method, Then, the total sample size was allocated to each department proportional to the number of female students in the department. Secondly, participant students were selected from each department proportional to their year of study using simple random sampling technique.

The number of study participants from the selected departments was determined using probability proportionate-to-population size allocation methods depending on their educational year.

The data were collected using a pre-tested structured self administered questionnaire which adapted to the local situations (1, 2, 8). The questionnaire were prepared in English and translated in to Amharic and back retranslated to English to check its consistency.

The question on behavioral variables were measured with five point likert scale ranging from 1 (strongly disagree) to 5 (strongly agree), and mean scores for each construct was computed and dichotomized in to positive and negative. Respondents who scored above the mean were labeled as having positive attitude and those who scored below the mean were labeled as having negative attitude. This scoring was subsequently reversed for negatively stated statement, so that the higher the score, the stronger the positive construct. Knowledge- questions were calculated after a number of question were presented. Correct answers were given score 1 and incorrect answering 0. The sum was computed and those who scored above the mean were labeled as having “good” knowledge and those who scored below the average were labeled to have “Poor knowledge'.

The respondents' attitude were measured using statements such as: (i) Emergency contraceptives use will not cause infertility in women (ii) Using emergency contraceptives after unsafe sexual intercourse is helpful, (iii) Emergency contraceptives creates lack of confidence between regular partners, (iv) it is good idea to avail emergency contraceptives for all females (v) it is sin to use emergency contraceptives methods (vi) The service of EC in campus or nearby clinic is convenient.

Data collection facilitators were recruited from Adama University instructors, who had BSc and representatives of students were used as data collectors after training on the study objective and instruments. Data were cleaned, coded and analyzed using SPSS versions 16.0. Logistic regression analysis was used to evaluate the association between different variables and knowledge, attitude and practice of participants about EC. P-value less than 0.05 at 95% CI was taken for statistical significance.

Ethical clearance and approval was obtained from ethical clearance committee of Faculty of Public Health, Jimma University after submission of the proposal, and a written consent was obtained from Adama University. All the study participants were informed about the purpose of the study, and their consent was obtained.

## Results

A total of 660 students completed the questionnaire making a response rate of 100%. Age of study participants ranged from 18–31 years with mean of 20.2 ± 1.7 years. Most, 468 (70.9%) of the respondents were followers of the Orthodox Christianity followed by protestant and Muslim which accounted for 95 (14.4%) and 86(13.0%), respectively. Five hundred and ninety seven (90.5%) of the respondents were single (table 1).

**Table 1:** Socio-demographic characteristics of female students, Adama University, Ethiopia, February, 2009.

Socio demographic characteristics	Number	Percent
**Age (n=660)**		
15–19	220	33.3
20–24	427	64.7
25–29	7	1.1
>30	6	0.9
**Year of study(n=660)**		
Year I	315	47.7
Year II	247	37.5
Year III	98	14.8
**Residence(n=660)**		
Campus	610	92.4
Out of Campus	50	7.6
**Religion(n=660)**		
Orthodox	468	70.9
Muslim	86	13.0
Protestant	95	14.4
Catholic	7	1.1
Others	4	0.6
**Marital status(n=660)**		
Single	597	90.5
Married	61	9.2
Divorced	2	0.3
**Number of children(n=660)**		
None	629	95.3
One	24	3.6
Two	7	1.1

On 194 (29.4%) of the respondents who were sexually active, 63(32.5%) students had an experience of pregnancy at least once Majority, 58(92%) of the pregnancies were unwanted and (77.7%) of pregnancies were ended with induced abortions. Six hundred twenty eight (95.2%) respondents have heard about regular modern contraceptive methods. Oral contraceptive pills were the most commonly known method 581 (92.5%) followed by injectables 497 (79.1%) and condoms 450(71.7%). Mass media for 288(45.9%), schools teachers for 271 (43.2%), health workers for 224 (35.7%), school clubs and reading articles for 205 (32.7%), for 117(18.6%) friends/and peers and parents for 84(13.4%) were the common source of information. Out of the total 67 (10.7%) students who ever used modern contraception, 34.5% were sexually active (table 2).

**Table 2:** Reproductive History and contraceptive practice of Female Students of Adama University, Ethiopia, February, 2009.

Variable	Number	Percent
**Sexually Active (n= 660)**		
Yes	194	29.4
No	466	70.6
**Age of first sexual intercourse (n = 194)**		
Younger than 10 years	7	3.6
10–14 years	30	15.5
15–19 years	144	74.2
20+ years	13	6.7
**Ever been pregnant (n=194)**		
Yes	63	32.5
No	131	67.5
**Unwanted pregnancy (n=63)**		
Yes	58	92.1
No	5	7.9
**Reason for unwanted pregnancy (n=58)**		
Contraceptive failure	8	13.8
Abandoned (pressure) by partner	3	5.2
Rape(Forced to have sex)	11	18.96
Rapture of condom	7	12.0
Forget to take contraceptive	24	41.4
Lack of knowledge about EC	5	8.6
**Experience of induced abortion (n=58)**		
Yes	49	84.5
No	7	12.1
I don't know	2	3.4
**Place of induced abortion (n=49)**		
Self infliction	7	14.3
Clinics	15	30.6
Untrained abortionist	27	55.1
Reason to have induced abortion(n=49)*		
Fear of parents and family	16	32.7
Fear of discontinuing school	33	67.3
Economic problems	6	12.2
**Ever heard about modern contraceptive (N=660)**		
Yes	628	95.2
No	32	4.8
**Ever used modern contraceptives (n=628)**		
Yes	67	10.7
No	561	89.3

* Multiple responses

Of the total participants, 309 (46.8%) ever heard about EC; the sources of information were peers/friends 148(47.7%), schools club 80 (25.8%), mass media 66(21.3%) and health workers 42(13.5%). When asked about specific types of emergency contraceptives, among those who have ever heard of EC, only 135 (43.7%) and 16 (5.2%) identified correctly emergency contraception pills (ECPs) (Progestin only pill and combined oral contraceptive) and intra uterine devices (IUDs) respectively as emergency contraceptive methods. Among those who were aware of emergency contraception, only 59(19.1%) and 11(3.5%) correctly identified 72 hours and 120 hours as the time limit for the ECPs and IUCD, respectively. When asked about the indication for EC, only 43(13.9%) mentioned the correct indication (after unprotected sex) while the rest gave different incorrect responses including after unwanted pregnancy by 76(24.2%), use as on going regular contraceptive by 101(33.9%) and do not know by 89 (28.4%). One hundred and sixty eight (54%) respondents stated that they could get EC from pharmacy, 132 (42.7%) from government hospitals and health centers and 59 (19%) of them said they could from private health institutions. When an overall knowledge score was computed, only 84 (27.2%) had good knowledge while 225(72.8%) had poor knowledge about the method. Although 415(62.9%) participants had positive attitude towards emergency contraceptives, the prevalence of ever use of emergency contraception among was only 4.7%. Emergency contraceptive pills were the commonest EC method used which accounted for 23(74.2%) (table 3).

**Table 3:** Knowledge, attitude and practice about emergency contraceptives among female university students; Adama University, Ethiopia, February, 2009.

Variables	Number	Percent
**Ever heard about EC N= 660**		
Yes	309	46.8
No	351	53.2
Methods reported as EC (N=309)*		
OCP	135	43.7
IUCD	16	5.2
Incorrect methods	262	71.8
**Indication/Circumstance EC used (n=309)**		
After unprotected sex	43	13.9
When unwanted pregnancy occurs	76	24.2
As ongoing MC	101	33.9
I don't know	89	28.4
**Time EC can work (n=309)**		
**ECP**		
With in 72 hrs	59	19.1
With in 120 hrs/5days	55	17.8
I don't know	195	63.1
**IUCD**		
With in 72 hrs	54	17.5
With in 120 hrs/5days	11	3.5
I don't know	244	79
Source of EC (n=309)*		
Pharmacy	168	54.4
Private clinic/youth center	59	19.1
Gov't institution	132	42.7
Shop	3	1
I don't know	9	2.9
**Knowledge of EC (n=309)**		
Poor(not good) Knowledge	225	72.8
Good Knowledge	84	27.2
**Attitude EC (N = 660)**		
Positive	415	62.9
Negative	245	37.1
**Ever Used EC (n= 194)**		
Yes	Yes	Yes
No	No	No

* Multiple responses

To determine the association between dependent variables and EC Knowledge, attitude and practice; bivariate and multiple logistic regression analyses were done. After controlling other variables, students of age 20 and above were more likely to have knowledge of EC than their counter parts (under 20 years) AOR=1.3; 95% CI: 1.18, 2.37. Moreover, as the year of study in campus increases, there appears to be a relative increase on emergency contraceptive knowledge AOR= 1.5; 95%CI: 1.12, 2.83 for year two, and AOR=0.97; 95%CI 0.55–1.75 for year three students. Female students who had sexual intercourse were found 4.9 times more likely to be aware of EC than their counter parts AOR= 4.9; 95%CI: 2.68, 9.65. After adjusting for other variables, positive attitude towards EC was significantly higher among the respondents who had ever used regular contraceptives than those who had no experience it (AOR= 2.98; 95% CI: 3.47, 5.76). Besides, positive attitude towards emergency contraceptives was higher among Protestants compared to Orthodox Christians and Muslims (AOR=1.6; 95% CI,:1.31, 2.73), and among senior students compared to their juniors (AOR=1.89; 95% CI: 1.43, 3.10). The likelihood of favorable attitude towards EC among those female students who had sexual intercourse was six times higher than their counter parts (AOR= 6.8; 95%CI: 3.80, 7.45) (table 4).

**Table 4:** Factors associated with Knowledge and attitude of EC among Adama University female students; Ethiopia; February 2009.

	Knowledge		Attitude		Practice	
**Variables**	**Crude OR** **(95% CI)**	**Adjusted** **OR(95% CI)**	**Crude OR** **(95% CI)**	**adjusted OR** **(95% CI)**	**Crude OR** **(95% CI)**	**adjusted OR** **(95% CI)**
**Age**						
15–19 years	1.00	1.00	1.00	1.00	1.00	1.00
20+&above	2.1(1.32,2.84)	1.3(1.18,2.37)	2.7(1.88,3.64)*	1.9(2.2,4.96)*	3.5(1.802,10.1)	2.37(1.102,7.25)*
**year of study**						
year one	1.00	1.00	1.00	1.00	1.00	1.00
year two	2.2(1.56,3.28)	1.5(1.12,2.83)*	1.2(0.56,2.68)	1.5(0.84,3.67)	3.25(1.33,7.97)	3.15(0.944,10.49)
year three	1.2(1.08, 1.97)	0.97 (0.55, 1.75)	2.6 (1.78, 3.6)	1.9(1.43, 3.10)*	3.4(1.157,9.901)	3.88(0.905,16.64)
**Religion**						
Orthodox	1.00	1.00	1.00	1.00	1.00	1.00
Muslim	0.65(0.82, 1.8)	0.55(0.37,1.32)	1.7(0.89,2.23)	1.43(0.7,1.86)	1.31(0.404,3.53)	0.176(0.080,1.86)
Protestant	1.65(1.87,3.6)	0.96(0.76,1.81)	1.8(1.42,2.86)*	1.6(1.31, 2.73)*	4.1(1.762,9.32)	1.19(0.762,5.417)
**Marital status**						
Never married	1.00	1.00	1.00	1.00	1.00	1.00
married	1.56(1.36,3.71)	1.23 (0.81, 2.13)	1.4(0.77,2.92)	1.23(0.67,2.26)	15.4(7.14,33.19)	9.3(2.538,20.73)*
**Number of** **children**						
None	1.00	1.00	1.00	1.00	1.00	1.00
One and above	0.8(0.16,1.51)	0.59(0.14,1.54)	1.38(0.41,2.71)	1.25(0.51,2.31)	9.16(3.7,22.67)	4.1(0.99,13.82)
**Sexual** **experience**						
No	1.00	1.00	1.00	1.00	1.00	1.00
yes	7.38(3.68,11.1)	4.9(2.68, 9.65)*	6.20(2.47,8.98)	4.8(3.80, 7.45)	5.68(2.48,13.1	3.32(0.78,10.6
**Ever used RC**						
No	1.00	1.00	1.00	1.00	1.00	1.00
Yes	2.6(3.2, 6.78)	1.8(0.42, 5.56)	2.6(3.2, 6.78)	2.98(3.47,5.76)*	3.3(1.49,7.21)	1.653(0.72,6.345)

* indicate statistical significant at P<0.05

When controlled for possible confounding effects of other covariates that showed association with emergency contraceptives practice in bivariate analysis, study subjects who were married subjects were nine times more likely to use EC than singles (AOR=9.3; 95%CI: 2.54, 20.73). On the other hand, the likelihood of using EC was twice higher among students aged ≥20 years than those younger (15–19 years) (AOR=2.37; 95%CI: 1.102, 7.25) (table 4).

## Discussion

In this study almost one-third of the subjects reported that they are already sexually active. This result is similar to the study conducted among college students in Assella (6). The result is also higher than similar study conducted on higher education students in Addis Ababa (8). But it was lower than the finding of studies conducted on South Africa Secondary School female students and Nigerian female undergraduates' students (1, 9).

In this study nearly one-third of ever sexually active respondents gave history of at least one pregnancy, of which 92% were unwanted pregnancies. But, the prevalence of unwanted pregnancy among the total study participants was 8.8 %, which is lower than reported by other studies conducted in the country, which ranged between 15–50% (4, 8, 11).

This study showed overall low rate of induced abortion. Of those with induced abortions, nearly two-third inflicted themselves and almost one-six by untrained abortionists. Similar Study conducted in Addis Ababa showed higher rate of unwanted pregnancy (73.5%), high rate of induced abortion (71.7%) and lower rate of safe abortion (29%) (8). The possible explanation for low rate of safe abortion and high rate of unwanted pregnancy in this study could be attributed to fear of parents and family, and economic problems made the respondents to take measures that could threaten their life or darken their future career.

Less than one-third of the respondents had good knowledge about EC on overall summary index for knowledge in this study which is comparable with studies conducted in other parts of Ethiopia, Nigeria, and Cameron (8, 9, 13). This finding reveals that comprehensive knowledge about EC methods is lacking among university female students.

The positive attitude of respondents towards EC is slightly higher than report from Addis Ababa (8) though a considerable proportion reported their concern on using it. Majority of the respondents got the service from pharmacy and only less than a quarter from government institution which is similar to the study done in Uganda (2).

This study showed that use of EC is low which is similar to the findings of studies in different parts of Ethiopia (8,10) but less than the report from studies conducted in South Africa and Nigeria (1, 9). The low EC practice rate in this study could be due to the fact that less proportion (29%) of them were sexually active compared to the report from South Africa and Nigeria where 57% and 63%, respectively were sexually active. The low awareness about EC could contribute. The fact that this study was institution-based; our findings might not be generalized to the general population. However, it may represent female students of higher learning institutions of Ethiopia.

The ever use of contraceptive in this study was comparable to a study conducted among South Africa Secondary school students (11%) and Addis Ababa Higher Education students (10%) and Ethiopian DHS (17.4%) (1,8,12).

Though nearly half of the respondents have heard about EC in this study, only 15% of them had identified the correct timing of administration of the pills after unexpected sexual contact. Several studies conducted in higher institutes in Uganda, Nigeria, South Africa, Cameron, and other developing countries reported similar findings (2, 9,13).

The main sources of information about EC were peers/friends, schools clubs and mass media which is similar with the finding in Uganda and Assella, Ethiopia and Addis Ababa, Ethiopia (2, 6, 8). In agreement with other studies findings, oral EC pill and IUCD were the most widely known and used emergency contraception (5,14).

In conclusion, in the presence of an increased risk of unwanted pregnancy and induced abortion among the sexually active students, the knowledge, and practice on emergency contraceptive was very low. Based on the findings, it is crucial to develop a strategy to increase awareness, knowledge, positive attitude, need based practice of emergency contraceptives and decrease barriers among respondents.

## Figures and Tables

**Figure 1:**
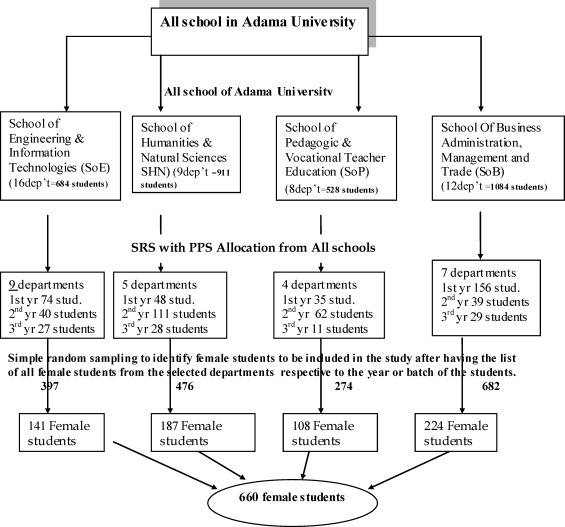
Schematic presentation of sampling procedure.

## References

[1] Manena-Netshikweta Ml (2007). Knowledge, perception and attitude regarding contraceptives among secondary school learners in the Limpopo province.

[2] Byamugisha Josaphat Kayogoza (2007). User and provider perspectives emergency contraception among young people in Uganda.

[3] Larsson Margarenta (2004). The Adoption of a New Contraceptives methods- surveys and intervention regarding emergency contraception. Acta Universitatis Upsalaliensis.

[4] Tadesse E, Yoseph S, Gossa A (1994). Illegal abortion in five hospitals in Addis Ababa. Ethiop Med J.

[5] Consortium on Reproductive Health Association (CORHA) (2005). Assessment of the reproductive health situations/ problems of students in the Addis Ababa, Bahirdar, Jimma, and Mekele Universities.

[6] Seife M, Fikre E (2007). Assessment of level of awareness and utilization of emergency contraception, among college female students in Oromia Regional state, Arsi Zone, Asella, South-East Ethiopia.

[7] Ethiopian Society of Obstetricians and Gynecologists (ESOG), Ministry of Health (FMOH) and ECafrique (2005). A training curriculum for mid-level health workers in Ethiopia. News letter.

[8] Wegene T, Fikre E (2005). Knowledge, attitude and practice on Emergency Contraceptives among female students at higher educations in Addis Ababa. Ethiop J Health Dev.

[9] Aziken Michael E, Okonta Patrick I, Ande Adedapo BA (2003). Knowledge and Perception of Emergency Contraception among Female Nigerian Undergraduates. International Family Planning Perspectives.

[10] Zeleke G, Zebenay Z, Weldegerima B (2009). Knowledge Attitude and Practice of Emergency Contraceptives in Bahir Dar University Female Students. Ethiopian Journal of Reproductive Health.

[11] Fantahun M, Chala F, Lola M (1995). knowledge, Attitude and practice of family planning among high school students in north Gonder. Ethiop Med J.

[12] Central statistics authority (CSA) (2005). Ethiopian Demographic and Health Survey.

[13] Eugene J, Pius N, Nelson F (2007). A survey of knowledge, attitudes and practice of emergency contraception among university students in Cameroon. BMC Emergency Medicine.

[14] Kumbi Solomon (2006). Emergency contraceptive now available in five regions in Ethiopia.

[15] Sorhaindo A, Becker D, Fletcher H, Garcia SG (2002). Contraception, emergency contraception among university students in Kingston, Jamaica: A survey of knowledge, attitude and practice. Middle East Fertility Society Journal.

